# Selective Laser Trabeculoplasty: An Overview

**DOI:** 10.5005/jp-journals-10008-1111

**Published:** 2012-08-16

**Authors:** Bhaskar Jha, Shibal Bhartiya, Reetika Sharma, Tarun Arora, Tanuj Dada

**Affiliations:** 1 Dr RP Centre for Ophthalmic Sciences, All India Institute of Medical Sciences, New Delhi, India; 2 Consultant, Glaucoma Services, Eye 7 Group of Hospitals, New Delhi India; 3 Dr RP Centre for Ophthalmic Sciences, All India Institute of Medical Sciences, New Delhi, India; 4 Dr RP Centre for Ophthalmic Sciences, All India Institute of Medical Sciences, New Delhi, India; 5 Dr RP Centre for Ophthalmic Sciences, All India Institute of Medical Sciences, New Delhi, India

**Keywords:** Selective laser trabeculoplasty, Lasers in glaucoma, Argon laser trabeculoplasty, Compliance and glaucoma.

## Abstract

Given the obvious quality of life concerns with medical and surgical lowering of intraocular pressure (IOP), lasers have received considerable attention as a therapeutic modality for glaucoma.

Selective laser trabeculoplasty (SLT) is increasingly being used in clinical practice as both the primary procedure and as an adjunct to medical and surgical therapy. Preliminary published evidence suggests that SLT is an effective, compliance-free, repeatable and safe therapeutic modality having only minor, transient, self-limiting or easily controlled side effects with no sequelae.

This review attempts a broad overview of the current knowledge of its mechanism, efficacy, indications and limitations, point out the knowledge lacunae that still exist with respect to this highly promising technology which has captured the attention of glaucoma surgeons all over the world.

**How to cite this article:**

Jha B, Bhartiya S, Sharma R, Arora T, Dada T. Selective Laser Trabeculoplasty: An Overview. J Current Glau Prac 2012;6(2):79-90.

## INTRODUCTION

Glaucoma is a progressive optic neuropathy and may lead to irreversible blindness. It is one of the leading causes of blindness, worldwide.^1^ The only known modifiable risk factor for disease onset and progression is intraocular pressure (IOP), the reduction of which may slow down progression in glaucoma.

Given the obvious quality of life concerns with medical and surgical lowering of IOP, lasers have received considerable attention as a therapeutic modality for glaucoma.

Laser application to the trabecular meshwork (TM) or laser trabeculoplasty (LTP) has been used since the early 1970s. It was first described in 1973 by Worthen and Wickham,^2^ using an argon laser to perform trabeculoplasty, and by Krasnov^3^ using a Q-switched ruby laser to perform goniopuncture or laser puncture. It was not until 1979 that Wise and Witter^4^ described a modified technique to perform argon laser trabeculoplasty (ALT), which subsequently gained acceptance as a therapeutic option of open-angle glaucoma (OAG). Since then, it has been seen that trabeculoplasty can also be performed with Krypton lasers,^5^ continuous wave neodymium lasers^6^ and diode lasers^7^ with results similar to those seen with ALT.

In the glaucoma laser trial follow-up study, after 7 years of follow-up, patients with ALT had lower IOP than patients on medical treatment (the glaucoma laser trial research group 1990, 1995).^8,9^ LTP appears to be less costly than current medical treatment.^10^ LTP can lower IOP without relying on patient compliance with medications, which has been repeatedly shown to be a common problem.^11-14^

By replacing or decreasing the need for topical medications, LTP can reduce systemic side effects, such as cardiorespiratory depression^15^ and local side effects, such as allergy,^16^ chronic inflammation^17,18^ which may decrease the success rate of future filtration surgery^19^ and cosmetic changes.^20^

Selective laser trabeculoplasty (SLT) was listed by the FDA in 2002 as a procedure separate from ALT. SLT is the delivery of laser energy to TM using parameters such that the result is the selective absorption of energy by pigmented cells, sparing adjacent cells and tissues from thermal damage. Although the mechanism of its action is even less well understood than that of ALT, it provides IOP reduction that is similar in magnitude and duration to that obtained with ALT, is associated with very few complications and offers several other potential advantages over ALT.

This review attempts a broad overview of the current knowledge of its mechanism, efficacy, indications and limitations point out the knowledge lacunae that still exist with respect to this highly promising technology which has captured the attention of glaucoma surgeons all over the world.

## MECHANISM OF ACTION OF SLT

This procedure is called SLT because there is a selective effect on melanotic elements associated with the meshwork. SLT is possible because pigmented TM cells exhibit greater optical absorbance of the applied laser energy than the cells that surround them.^21^ Histologic studies have shown that there is less coagulative damage after SLT and less structural change of the meshwork.^22^ A short burst of laser energy heats and thermally damages pigmented TM cells before neighboring cells have a chance to absorb enough laser energy to incur any thermal damage.^23^ Latina and Park have demonstrated selective killing of the cultured, pigmented TM cells over nonpigmented TM cells using the principles of selective photothermolysis (SP).^23-26^ The pigmented TM endothelial cells have melanin as the intracellular pigment and, thus, it is possible to confine the damage to these cells using the principles of SP. This approach relies on selective targeting of the pigmented TM cells using single laser pulses of short pulse duration and low fluencies (energy/area), to generate and confine heat to the pigmented cells.^23^ One important characteristic of the principle of SP is that multiple targeting of only the pigmented TM cells can be affected simultaneously without using focusing to achieve specificity. Using a short laser pulse confines the thermal damage to the cell containing the pigment target, if the duration of the laser pulse is equal to or less than the thermal relaxation time of the intracellular pigment.^23,27^ The thermal relaxation time of melanin is approximately 1 millisecond and the pulse duration of SLT laser is 3 nanosecond, and this essentially prevents thermal dissipation to surrounding tissues.^27^ The large spot size in SLT (400 mm) versus the small spot size in ALT (50 mm) also ensures that low fluences can be maintained, which is necessary to prevent collateral thermal damage to the surrounding tissues.^23^ Although melanin has a broad absorption spectrum, it has been shown that for the destruction of melanin-containing cells, a wavelength of about 504 nm seems optimal.^23,28^ The mechanism of action of SLT has been explained by biological theory which suggests that trabecular photocoagulation stimulates some biologic response that could contribute to eventual reduction in the IOP.^29^ Although ALT destroys both the pigmented and the nonpigmented TM endothelial cells at the burn site, it has been shown in studies using human TM maintained in organ culture model that, after ALT, the trabecular cells in the region near the Schwalbe line are stimulated to divide.^30,31^ This cell division seemed to occur not only in the half treated with laser photocoagulation but also in the opposite 180° nonlasered TM segments. No stimulation of the corneal endothelial cells or trabecular overgrowth by these cells was noted in these studies. It has been suggested that perhaps these cells in the anterior nonfiltering portion of the TM are multipotent cells, and that injury to the trabeculum from laser trabeculoplasty stimulates them to divide, to migrate and repopulate the TM beams at the burn sites, to fabricate a new extracellular matrix or phagocytize.^32^ Although the precise barriers to aqueous humor outflow are not understood, it has been suggested that the proteoglycan components of the extracellular matrix are significant contributors.^33,34^ It has been shown that argon laser photocoagulation of the TM causes alteration in the turnover or synthesis of trabecular extracellular matrix and this might result in an improvement in the aqueous outflow.^35^ The stimulation of the trabecular cells with an increase in their phagocytic activity, after the ALT, might also help in cleaning the TM of any ‘clogging’ debris.^36^

### Predictive Factors of SLT Success

The predictors of success following SLT have been studied in various population, and the results obtained have been found to be similar, though not identical. This may be attributed to the fact that the population studied varied in terms of race, age, severity of glaucoma and also SLT treatment protocols (Table 1).

Gracner et al^37^ reported a negative correlation between successful SLT and the grade of TM pigmentation and diabetes. They did not find any significant correlations between successful SLT and baseline IOP, age, gender, hypertension, family history of glaucoma, previous anterior segment surgery, laser energy used or local antiglaucoma therapy. They found that SLT efficacy is positively associated with IOP elevation before laser treatment. This result is consistent with Hodge et al^38^ who reported that SLT success was significantly predicted by baseline IOP but not by age, sex, other glaucoma risk factors, type of open angle glaucoma or by degree of TM pigmentation.

**Table 1:** **Table 1:** Predictors of success following SLT

*Study*		*Population*		*Treatment*		*Follow-up duration*		*Negative correlation*		*Positive correlation*		*No significant relation*	
Gracner et al^37^								Diabetes, trabecular meshwork pigmentation		IOP elevation before laser treatment		Baseline IOP, age, sex, hypertension, family history of glaucoma, previous anterior segment surgery, laser energy used and local antiglaucoma therapy	
Hodge et al^38^										Baseline IOP		Age, sex, other glaucoma risk factors, type of open angle glaucoma or by degree of trabecular meshwork pigmentation	
Mao et al^39^								IOP before SLT treatment		IOP before SLT treatment		Similar correlation	

Mao et al^39^ reported a positive association between laser efficacy and IOP before SLT treatment. Marcelo et al^40^ conducted a retrospective review of reports of 120 eyes which had SLT, and found a significant correlation between successful SLT and age, baseline IOP and amount of laser energy delivered.

### Clinical Results of SLT

Preliminary clinical results with SLT were reported by Latina et al^41^ in 1998 in a study of 53 eyes of patients with open angle glaucoma (OAG) and uncontrolled IOP on maximum tolerated medical therapy (MTMT) or after previous ALT. Nearly all eyes had primary OAG (POAG). The nasal 180° were treated. Follow-up ranged from 4 to 26 weeks, with 44 eyes completing 26 weeks of follow-up. IOP was reduced, on average by 4.1 mm Hg (16.3%) at 1 week and by 4.6 mm Hg (18.7%) at 26 weeks. IOP in the untreated eye at 26 weeks was reduced by 2.1 mm Hg (9.7%). At 26 weeks, IOP had decreased by more than 3 mm Hg and by a mean of 5.9 mm Hg (23.8%) in 32 eyes. Eyes were not treated with glaucoma medications immediately prior to the procedure, and IOP elevation of greater than 5 mm Hg was observed in 13 eyes (25%). In all cases, this was seen within 2 hours and resolved with topical antihypertensive medications within 24 hours. Mild-to-moderate anterior chamber reaction was observed in 44 eyes (83%) one hour after the procedure, and resolved completely within 5 days in all cases with routine postoperative treatment of prednisolone acetate 1% four times daily for 5 days. Except for transient pain and blurred vision in 15% of cases and redness in 9%, no other complications were reported.

Lanzetta et al^42^ reported that 360° SLT could achieve clinically significant IOP reduction in eight eyes with high IOP that was uncontrolled with MTMT, even after previous ALT or trabeculectomy. Lower IOP that was observed after 24 hours had remained stable through 6 weeks. One eye had an immediate postoperative IOP rise of 10 mm Hg, which dissipated quickly.

Chen et al^43^ prospectively compared SLT of 90° and 180° in two groups of 32 patients with ocular hypertension (OHTN) or primary, pigmentary or exfoliative OAG. Some eyes had previously undergone ALT. At 7 months follow-up, 13 of 32 eyes and 15 of 32 eyes in the two groups respectively, required retreatment or trabeculectomy. Excluding these eyes, mean IOP was reduced from 25.44-1.41 to 18.43-1.38 mm Hg and from 26.06-1.73 to 19.90-1.59 mm Hg by each treatment respectively, with the difference not statistically different. No acute or delayed complications were reported. The degree of trabecular pigmentation was significantly related to the degree of IOP reduction at 7 months but not at 1 or 4 months. Throughout follow-up, neither the presence of exfoliation or previous ALT treatment was significantly related to IOP reduction throughout the follow-up.

A study by Melamed^44^ et al suggests that SLT may be a safe, noninvasive, and effective treatment modality in OAG as a primary treatment instead of topical medications or ALT. Of 31 patients with POAG or OHT, 45 eyes underwent SLT as primary treatment. The mean ± SD baseline IOP for all eyes was 25.5 ± 2.5 mm Hg and the final IOP was 17.9 ± 2.8 mm Hg, a decrease of 30%. Only three eyes required topical ocular hypotensive medications to reduce IOP post-SLT. Most patients (40 eyes or 89%) had an IOP decrease than 5 mm Hg at the end of the follow-up period (18 months).

The results of the main studies are summarized in Table 2.

## COMPARING MEDICAL MANAGEMENT AND SLT

L Jay Katz et al^49^ conducted a prospective randomized clinical trial to compare outcomes SLT of with drug therapy for glaucoma patients in 69 patients (127 eyes) with open angle glaucoma or ocular hypertension. Target IOP was determined using the collaborative initial glaucoma treatment study formula. Patients were treated with SLT (100 applications 360°) or medical therapy (prostaglandin analog). Six visits over 1 year followed initial treatment. Twenty-nine patients were in the SLT group, 25 patients in the medical group. Baseline mean IOP for all eyes was 24.5 mm Hg in the SLT group, 24.7 mm Hg in the medical group. Mean IOP (both eyes) at last follow-up was 18.2 mm Hg (6.3 mm Hg reduction) in the SLT arm, 17.7 mm Hg (7.0 mm Hg reduction) in the medical arm. By last follow-up, 11% of eyes received additional SLT, 27% required additional medication. There was no statistically significant difference between the SLT and medication groups.

**Table 2:** **Table 2:** Clinical results of SLT

*Study*		*Population*		*Treatment*		*Baseline IOP*		*Follow-up*		*IOP reduction*		*Definition of success*		*Response rate*		*Comments*	
Latina et al^41^		53 eyes with OAG on MTMT with ALT; 44 eyes 26 weeks follow-up was done		SLT nasal 180°		24.6		26 weeks		4.6 mm Hg (18.7%)		IOP reduction >3 mm Hg		73%			
Lanzetta et al^42^		8 eyes of 6 patients with POAG on MTMT some with previous ALT		360°SLT		26.6		6 weeks		10.6 ± 5.5 mm Hg (40%)		NA		NA		NA	
Kajiya et al^47^		17 eyes of 10 patients with POAG 1 eye with XFG		180°SLT		22.8		6 months		6.7 mm Hg		NA		NA			
Chen et al^43^		2 groups of 32 patients with or OAG some with previous ALT		180°		26.06 ± 1.73 mm Hg		7 months		6.16 mm Hg		IOP controlled without retreatment or trabeculec-tomy		59%		IOP reduction positively correlated with trabecular meshwork pigmentation	
Johnson et al^44^		132 eyes (95 patients) with OAG		360°SLT		20.9		3 months		3.74-4.58 mm Hg (12.4%)		IOP reduction $ 30%		24%			
Cvenkel^45^		44 eyes of 31 patients with medically controlled OAG		SLT inferior 180°		25.57 (range 22-34)		12 months (40 eyes)		4.8 mm Hg (18.6%) at 6 months 4.4 mm Hg (17.1%) at 12 months		IOP reduction $ 3 mm Hg		66% at 3 months			
Gracner^46^		50 eyes with OAG		180° SLT		22.48		6 months		5.06 ± 2.37 mm Hg		IOP reduction >3 mm Hg 60%		88%			
Hodge et al^48^		72 patients with OAG on MTMT		180°SLT 23.8 ± 4.88		12 months		5.8 mm Hg		IOP reduction of >20% after 1 year						IOP reduction significantly related to baseline IOP	
Melamed et al^44^		45 eyes of 31 patients with OAG or OHTN 37 newly diagnosed		Nasal180° SLT		25.5 ± SLT		Range of 3-24		7.7 ± 3.5 at last follow-up		IOP reduction >20%		96%			

Nagar et al^50^ conducted a prospective, randomized trial in which 167 eyes of 167 subjects received either 90, 180 or 360 degrees SLT or latanoprost 0.005% once daily at night. Successful treatment was defined as either >20% or >30% IOP reduction from baseline with no further IOP-lowering interventions. After a mean follow-up period of 10.3 months (range, 1-12 months), latanoprost therapy demonstrated superior success by both success definitions (90 and 78%, respectively) compared with either 90° (34 and 11%, respectively) or 180° SLT (65 and 48%, respectively), and compared with 360° SLT (82 and 59%, equivalent IOP success respectively).

Lai et al^51^ conducted a prospective, randomized trial in which 29 newly diagnosed subjects with ocular hypertension or OAG underwent 360° SLT in 1 eye and topical medical therapy (using 1 or more medications chosen by the investigator in a nonspecified fashion in the fellow eye). Mean IOP reduction after 5 years of follow-up was 8.6 mm Hg (32.1%) in SLT eyes and 8.7 mm Hg (33.2%) in medically treated eyes (p = 0.95); This included eight SLT-treated eyes (27.6%) that required medications after SLT to maintain IOP below 21 mm Hg. Treatment failure (IOP >21 mm Hg despite maximal medical therapy requiring filtering surgery) was observed in 17.2% of SLT eyes and 27.6% of medically treated eyes.

The results of the main clinical trials are summarized in Table 3.

### SLT Comparison to ALT

Damji et al^53^ prospectively performed 180° of SLT or ALT in a randomized fashion in 36 eyes of 34 patients with various forms of OAG. Some eyes had been previously treated with ALT. Average IOP and change in IOP did not differ statistically significantly between the two groups during 6 months of follow-up. At 6 months, IOP was reduced by 4.8 to 3.4 mm Hg in the SLT group and 4.7 to 3.3 mm Hg in the ALT group. Anterior chamber reaction was quantitated only 1 hour after the laser treatment, when there were, on average, statistically significantly more cells in eyes that had SLT and a similar amount of flare in both groups. These investigators subsequently extended their study to 176 eyes of 152 followed for 12 months. There was no statistically significant difference between IOP in the two groups at any time point from baseline to 12 months. At 12 months, IOP was reduced by a mean -5.86 to 6.1 mm Hg in the SLT group and - 6.0 to 4.8 mm Hg in the ALT group. Importantly, it should be realized that the results of this study reflect a ‘real-life’ scenario more than a controlled study, because medication change, additional laser treatment and surgery were allowed during the study period and are partly responsible for the resulting IOP, in addition to the initial laser.

Juzych et al^54^ conducted a retrospective analysis to compare the longer-term outcomes of SLT with ALT. They reviewed the charts of all patients with uncontrolled chronic OAG who were treated with laser trabeculoplasty over a 6-month period by the same surgeon. Of these patients, 154 were treated with ALT and 41 with SLT patients. Preoperatively, patients in both groups had a similar IOP (23.9-2.6 in SLT group, 24.3-4.1 in ALT group) been using a similar average number of glaucoma medications (2.5-1.3 in SLT group, 2.5-1.2 in ALT group). Follow-up ranged from 3 to 60 months (mean, 32.5-15.9 months). All eyes were pretreated with apraclonidine 1.0% and all underwent treatment of 180° of the TM. When success was defined as IOP reduction of at least 3 mm Hg without additional medications or surgery, it was recorded 1, 3 and 5 years after SLT in 68, 46 and 32% of patients and, after ALT, in 54, 30, and 31% respectively. Another criterion of success was defined as IOP reduction of 3 or more mm Hg and 20% or more of the pretreatment IOP. At 1, 3, and 5 years, this was observed in 58, 38 and 31% of patients after SLT and in 46, 23 and 13% after ALT respectively. The differences in success rates between SLT and ALT were not statistically significant.

Martinez-de-la-Casa et al^55^ prospectively performed SLT or ALT on the inferior 180° in two groups, each of 20 eyes with POAG and no previous ALT. The IOP was similarly reduced in both groups at all time points. In the SLT group, IOP was lowered from 24 to 4.7 mm Hg preoperatively to 22.1 to 3.7 and 18.6 mm Hg (22.5%) at 1 week and 6 months respectively. In the ALT group, it was lowered from 23.6 to 3.8 mm Hg preoperatively to 20.9 to 3.4 and 19-3.2 mm Hg (19.5%) at 1 week and 6 months respectively. An IOP reduction of at least 3 mm Hg was observed in 80% of the SLT-treated eyes (a mean percent decrease of 26.7%) and in 85% of ALT-treated eyes (a mean decrease of 21.8%).

The results of the main comparative studies are summarized in Table 4.

## HOW MUCH ANGLE IS TO BE TREATED?

The principle of therapy by any modality is to apply the minimum amount of treatment necessary to achieve the maximum desired therapeutic with minimum adverse effects. As proved by many studies, SLT has a dose response effect.

In the study by Nagar et al,^50^ 90° SLT treatment produced lower success rates than either 180 or 360° SLT; the higher success rates with 360° SLT compared with 180° SLT did not reach the level of statistical significance ocular pain but not anterior chamber inflammation or the rate of IOP spikes was increased in eyes receiving 360 *vs* 90° SLT.

Chen et al^43^ conducted a prospective randomized trial to compare two regimens of SLT. SLT with 25 laser spots on 90° of TM and SLT with 50 laser spots on 180° of TM in 64 subjects with uncontrolled OAG despite 2 or 3 IOP-lowering medications in a prospective. At 1, 4 and 7 months after treatment, mean IOP reduction (approximately 5-6 mm Hg) and the failure rate necessitating retreatment or incisional surgery (approximately 38% at 4 months and 45% at 7 months) were identical between the two groups. No adverse events were reported in either group.

**Table 3:** **Table 3:** Results of the main clinical trials

*Study*		*Population*		*Treatment*		*Baseline IOP*		*Follow-up*		*IOP reduction*		*Definition success*		*Response rate*	
Nagar et al^50^		167 patients with OHTN or OAG newly diagnosed or medically controlled after 8 washout		Xalatan		29.3		Mean 10.3 months (range 1-12)		NA		IOP reduction $ 20% IOP reduction $ 30% with no additional medications		90% 78%	
												IOP reduction $ 20%			
				90°SLT								IOP reduction $ 30% with no additional medications		34% 11%	
												SLT IOP reduction $ 20%			
				180°SLT								IOP reduction $ 30% with no additional medications		65%	
												IOP reduction $ 20%		48%	
												IOP reduction $ 30% with no additional medications		82%	
				360°SLT										59%	
Mcllraith et al^52^		100 eyes with newly diagnosed early OAG and OHTN		Latanoprost (26 eyes)		24.6		12 months		7.7 mm Hg (30.6%)		IOP reduction $ 30%		43%	
				SLT inferior 180° SLT (74 eyes)		26.0				8.3 mm Hg (31%)		IOP reduction $ 30%		55%	
Lai et al^51^		29 Chinese patients with POAG or OHTN, newly diagnosed		360 SLT (fellow eye treated with medication)		26.2-4.2		5 years (82.8% completed)		8.6-6.7 mm Hg (32.1%)		IOP #21 mm Hg without medication IOP #21 mm Hg on MTMT		72% 83%	
Jay Katz et al^49^		69 patients (127 eyes OAG or OHTN were randomized to SLT or medical therapy		SLT (100 applications 360°) or medical therapy (prostaglandin analog)		24.5 mm Hg 24.7 mm Hg in the medical group		9 to 12 months		(6.3 mm Hg reduction) in the SLT arm, (7.0 mm Hg reduction) in the medical arm					

Song et al^59^ conducted a retrospective chart review of SLT treatments; 94 eyes from 94 patients were included. A majority (83/92, 90%) underwent 180° SLT. Six months follow-up was done. SLT failure was defined in two ways: (1) IOP decrease, 3 mm Hg (definition one), or (2) IOP decrease 20% (definition two), on two successive visits 4 weeks after SLT. According to definition one, a total of 64 eyes (68%) failed. According to definition two, a total of 70 eyes (75%) failed. By the end of the study (14.5 months), the failure rates were 86 and 92% by definitions one and two respectively, overall failure rates of 68 to 74%.

Prasad et al^60^ conducted a retrospective chart review of patients to determine and compare the effect of 180 and 360° SLT treatment as a primary therapy on the intervisit intraocular pressure fluctuation in patients with ocular hypertension and primary open angle glaucoma who received SLT as primary therapy without any subsequent medical or surgical intervention followed up for a period of 2 years. Forty-one eyes were treated by SLT, 19 eyes in the 180° group and 22 eyes in the 360° group. The mean reduction in IOP at 2 years was 28% in 180° group and 35% in 360° SLT group. After the SLT, the 360° SLT group had a lower IOP fluctuation compared with the 180° SLT group over the follow-up period of months 6 to 24 months. This study suggests that 360° SLT is more efficacious in achieving smaller IOP fluctuations than treatment with 180° SLT.

**Table 4:** **Table 4:** SLT results over time

*Author*		*Type of study*		*Number* * of eyes*		*Duration of* * follow-up*		*IOP reduction* * in SLT*		*IOP reduction* *in ALT*		*p-value*	
Damji et al^53^		Prospective RCT		36		6 months		4.8 mm Hg		4.7 mm Hg		0.97	
Damji et al^56^		Prospective RCT		176		12 months		5.86 mm Hg		6.04 mm Hg		0.846	
Best et al^57^		Prospective RCT		165		12 months		1.8 mm Hg		2.1 mm Hg		NS	
Juzych et al^54^		Retrospective case series		195		37.4 months (SLT), 33.6 months (alt)				Percent with Z3 mm Hg reduction		Without further therapy	
Martinez-de-la-Casa et al^55^		Prospective RCT		40		6 months		18.6 mm Hg (baseline 24 mm Hg)		19.0 mm Hg (baseline 23.6 mm Hg)		0.81	
Popiela et al^58^		Prospective RCT		27 eyes		3 months		2.85 mm Hg		2.63 mm Hg		0.84	

**Table 5:** **Table 5:** Clinical results of SLT with respect of amount of angle treated

*Author*		*Method of study*		*Number of eyes*		*Degree of* * angle treated*		*Duration of* * follow-up*		*Conclusion*	
Prasad et al^60^		Retrospective chart review		41 (19 eyes in the 180° TM, 22 eyes in the 360° TM)		180 and 360° of TM		2 years		360° SLT is more efficacious	
Chen et al^43^		Prospective randomized trial		64		25 laser spots on 90° and 50 laser spots on 180° TM		1, 4 and 7 months after treatment		Identical between the 2 groups	
		Retrospective chart review		94 eyes		A majority (83/92, 90%) underwent 180° SLT		6 months		Failure rates of 68 to 74%	
Ayala et al^61^		Retrospective chart review		120 eyes				18 months		They recommend treating patients over 180° TM	

Ayala et al^61^ conducted a retrospective chart review of eyes that underwent SLT. The primary outcome measure was time to failure after SLT treatment, 120 eyes of 120 patients were identified. The average time to failure after SLT was 18 months. The success rate after 12 months was 62%, after 24 months 34%, after 36 months 28% and after 48 months 24%. The long-term effects of SLT, when eyes were treated over 90°, seem to be low. They recommend treating patients over 180°.

The results of the main clinical evaluations are summarized in Table 5.

## SLT IN DIFFERENT SUBTYPES OF GLAUCOMA

SLT is commonly used to treat patients with open angle glaucoma, (i.e. primary open-angle glaucoma, pigmentary glaucoma, exfoliative glaucoma). However, recent studies have examined the efficacy and safety of SLT to lower IOP in other glaucoma subtypes as well. Although limited by small sample sizes and lack of control groups, these studies suggest an expanding clinical role for LTP.

### SLT in PACG

SLT seems to be a safe and effective method of reducing IOP in many eyes with primary angle closure and a patent iridotomy in which there is a sufficient extent of visible TM. Ho et al^62^ conducted a multicentric, prospective, noncontrolled clinical trial to determine, whether SLT can lower intraocular pressure in eyes with chronic primary angle closure, elevated IOP, and a patent iridotomy. Sixty eyes of 60 patients with chronic angle closure who had undergone iridotomy, had an IOP greater than 21 mm Hg and a gonioscopically visible pigmented TM for at least 90° were enrolled. SLT was applied to open angle segments. Patients were followed-up for 6 months. The mean baseline IOP was 24.6 ± 2.5 mm Hg. At 6 months, IOP reduction of 3 or 4 mm Hg was measured in 82 and 72% of eyes respectively, and IOP reduction of 20 or 30% was measured in 54 and 24% of eyes respectively .There were no significant complications attributable to SLT.

### SLT in Steroid-induced Glaucoma

Steroid-induced glaucoma has become common with the rise in usage of intravitreal steroids to treat a variety of posterior segment disorders. There have been very few studies to assess the efficacy of SLT in steroid-induced glaucoma. SLT is a temporizing procedure to consider in patients with steroid induced elevated IOP.

Rubin^63^ et al conducted a retrospective review to access effectiveness of SLT in lowering IOP in patients with steroid-induced elevated IOP of seven patients (7 eyes) with IOP elevation after intravitreal triamcinolone acetonide (IVTA; 4.0 mg/0.1 ml) injections for macular edema (6 patients) or central retinal vein occlusion (1 patient). SLT lowered (p < 0.007) IOP in five eyes of seven patients with steroid-induced increased IOP from 3 weeks to 6 months postoperative.

Ercument et al^64^ conducted a prospective, comparative, interventional case series to evaluate the prophylactic efficacy of SLT for preventing an increase in IOP after IVTA injection, they studied 31 eyes with a baseline IOP of 21 mm Hg or more of 31 patients for which IVTA injection was planned for diabetic macular edema. The patients were divided into two groups. The study group comprised 15 eyes of 15 patients that underwent SLT a mean of 8.3 ± 4.1 days before IVTA injection. The control group comprised 16 eyes of 16 patients who underwent only IVTA injection. Main outcomes measures were mean IOP and number of patients requiring antiglaucoma therapy. They concluded that the IOP elevation after IVTA injection may be prevented by performing SLT before IVTA injection, especially in with a baseline IOP of 21 mm Hg or more.

Pizzimenti et al^65^ reported a case of steroid glaucoma induced by IVTA in which IOP declined from 38 mm Hg on maximal medical therapy to 16 mm Hg on no topical therapy within 2 months after 180° SLT.

### SLT in Pseudoexfoliative (XFG) Glaucoma

Gracner et al^66^ demonstrated in a small prospective trial consisting of 10 eyes each with POAG and XFG glaucoma that 180° SLT produced comparable IOP reductions in both groups. At 18 months, IOP was reduced by a mean of 35.1% in POAG eyes and 31.4% in XFG eyes.

Melamed et al^67^ included five eyes with XFG in which IOP was reduced by an average of 41%; because the sample size was small, these authors did not attempt statistical comparison to the eyes with POAG.

Chen et al^43^ found that after 1 and 4 months, the presence of XFG had no effect on post-SLT IOP reduction, but was significantly more prevalent in eyes that did not have retreatment, suggesting an association with increased success rate after SLT. One subgroup analysis showed that 1 year after SLT, XFG glaucoma was not associated with a different outcome compared with OAG. These preliminary findings suggest that SLT is effective in eyes with XFG, with similar efficacy to eyes with OAG and similar efficacy to that of ALT.

### SLT in Normal Tension Glaucoma (NTG)

El Mallah et al^68^ performed a retrospective study of SLT in 31 eyes of 18 patient diagnosed with NTG. The mean postoperative IOP was 12.2 ± 1.7 mm Hg from a baseline of 14.3 ± 2.6 mm Hg. SLT-treated eyes also showed decreased IOP fluctuation of 2.5 ± 1.9 (during 1-year posttreatment) *vs* 4.5 ± 2.5 mm Hg (pretreatment).

### SLT in Heavily Pigmented TM

There is some evidence showing a correlation between the degree of angle pigmentation and the effectiveness of SLT.

The study by Van de Veire et al^69^ comparing SLT to ALT included two eyes with pigmentary glaucoma who received SLT; both eyes experienced a paradoxical 16% rise in IOP. The same investigators then performed SLT with lower energy (<0.9 mJ) in six more eyes with heavily pigmented angles and observed a 19% rise in IOP that persisted for 12 weeks.

Harasymowycz et al^70^ reported IOP spikes in four patients with heavily pigmented angles, with peak IOPs ranging from 31 to 65 mm Hg; three of the four subjects required trabeculectomy.

Melamed et al^67^ included three cases of pigmentary glaucoma; in these patients, SLT produced an intraocular pressure reduction in 24% of eyes.

Damji et al^57^ obtained an intraocular pressure reduction of 5.6 mm Hg in five pigmentary glaucoma patients treated with SLT after 12 months.

### Adverse Effects of SLT

In general adverse effect in SLT have been transient and minor. Early postoperative elevation of IOP in some patients has been observed in all published series, whether or not the patients were receiving perioperative antihypertensive treatment. IOP can spike in 3 to 5% of eyes, commonly occurring 1 to 4 hours after the procedure.

IOP Spikes

Latina et al^48^ reported IOP spikes of 5 mm Hg or greater in 25% of SLT-treated eyes and IOP spikes of 8 mm Hg or greater in 9% of treated eyes. All manifested within 2 hours after treatment, resolved with IOP-lowering medications within 24 hours, and no eyes exhibited a persistent IOP elevation. Damji et al^53^ reported that 3.4% of ALT-treated eyes and 4.5% of SLT-treated eyes exhibited an IOP rise of 6 mm Hg or greater within 1 hour after treatment. Nagar et al^53^ reported that 27% of eyes undergoing 360° SLT manifested an IOP spike of 5 mm Hg or more (compared with no IOP spikes in latanoprost-treated eyes). Lai et al^51^ reported that 10.3% of360° SLT-treated eyes manifested an IOP spike of 5 mm Hg or greater.

Anterior Chamber Inflammation

In the study of 180° SLT by Latina et al,^48^ 83% of SLT-treated eyes exhibited mild-to-moderate inflammation, appearing within 1 hour after treatment, decreasing by 24 hours after treatment, and completely resolved in all cases within 5 days of treatment. Martinez-de-la-Casa et al^55^ evaluated flare using the Kowa flare meter and found significantly lower flare readings after SLT than ALT. Damji et al^53^ reported more anterior chamber inflammation in the first 1 hour after SLT than ALT. Nagar et al^50^ reported a 50% rate of anterior chamber inflammation in eyes receiving 360° SLT *vs* 0% in latanoprost-treated eyes. A single case report of hyphema after SLT has also been reported.

Ocular Discomfort

Latina et al^48^ reported that 15% of eyes receiving SLT reported discomfort after the procedure. Martinez-de-la-Casa et al^55^ evaluated postoperative pain using a 10-point scale and found significantly lower pain scores after SLT compared with ALT during and immediately after treatment; these differences were gone by 24 hours posttreatment. Nagar et al^50^ reported a 39% rate of discomfort in eyes undergoing 360° SLT and a 0% rate in eyes receiving latanoprost.

### Repeat Selective Laser Trabeculoplasty

SLT is becoming an increasingly popular alternative to ALT in the treatment of open-angle forms of glaucoma, primarily owing to the lack of collateral thermal damage and TM scarring with SLT as compared with ALT. Since SLT delivers only one tenth of the energy compare with ALT and it causes minimal thermal damage to TM it has a greater potential of repeatability. Since its approval, there have been very few studies to study the efficacy of repeatability of SLT despite of strong theoretical proof for it. Bryan Kun Hong et al^71^ conducted a retrospective chart review of forty-four eyes of 35 patients, underwent an initial 360° SLT (SLT1), which was successful for 6 months, but eventually lost efficacy and was followed by a repeat 360° SLT (SLT2). Using a definition of ‘success’ as >20% peak IOP reduction, success rates for SLT1 and SLT2 were not significantly different. They concluded that repeat 360° SLT may be safe and effective after an initially successful 360° SLT has failed.

### Comparative Analysis of Cost of Medical Management with SLT

There has been many studies to prove that the common cause of failure of medical management in glaucoma is lack of compliance. Nordstorm et al^72^ did a retrospective cohort study using health insurance claims data of newly treated individuals with diagnosed glaucoma (n = 3623) and suspect glaucoma (n = 1677), and concluded that many patients fail to use topical medications as prescribed. Nearly one half of the individuals who had filled a glaucoma prescription discontinued all topical ocular hypotensive therapy within 6 months, and just 37% of these individuals recently had refilled their initial medication at 3 years after the first dispensing .The other cause of failure of reported is cost of glaucoma medication.

Centaur et al^73^ compared the 5-year costs of three treatment strategies: medication, LTP, and filtering surgeries in managing patients with primary open-angle glaucoma whose intraocular pressures were not adequately controlled by two medication .The 5-year cumulative costs were approximately $6571, $4838 and $6363 for patients in the medication, LTP and filtering surgery arms respectively. Over 5 years, LTP was associated with the lowest total costs compared to treatment by medication alone or by filtering surgery for patients who were not adequately controlled by two medications.

Lee et al^10^ presented a projected 6-year cost comparison of primary SLT versus primary medical therapy in the treatment of open-angle glaucoma for patients aged 65 years or more their findings suggest that SLT as primary therapy, at a per-patient level, offers a modest potential cost saving over primary medical therapy in the management of open-angle glaucoma for patients aged 65 years or more. In the SLT rep 2y scenario, the use of primary SLT over mono-, bi-, and tri drug therapy produced a 6-year cumulative cost savings of 206.54 dollars, 1668.64 dollars, and 2992.67 dollars per patient respectively.

## CONCLUSION

SLT is increasingly being used in clinical practice as both, the primary procedure and as an adjunct to medical and surgical therapy. Preliminary published evidence suggests that SLT is a effective, compliance-free, repeatable and safe therapeutic modality having only minor, transient, self-limiting or easily controlled side effects with no sequelae. Also, the use of SLT has a better quality of life impact than medication or surgery. Its use has been found to be cost-saving, thereby reducing the economic burden of the disease.

However, the response rates within the first postoperative year have varied from 59 to 96%, according to different definitions. The reported average reduction in IOP from pretreatment IOP ranges from 18 to 40%, over a follow-up period of 6 to 12 months, with some authors reporting results for the whole cohort and others only for responders. Therefore, it must be emphasized that SLT is not a cure, and all patients must remain under regular follow-up.
